# L’arthroscopie de la hanche dans le conflit fémoro-acétabulaire: à propos de 2 observations

**DOI:** 10.11604/pamj.2018.30.36.14008

**Published:** 2018-05-17

**Authors:** Abdelhafid El Marfi, Badarou Chaibou, Kevin Parfait Bienvenue Bouhelo-Pam, Mohamed El Idrissi, Mohamed Shimi, Abdelhalim El Ibrahimi, Abdelmajid El Mrini

**Affiliations:** 1 Service de Chirurgie Ostéo-articulaire B4, CHU Hassan II, Université Sidi Mohammed Ben Abdellah, 3000 Fès, Maroc

**Keywords:** Arthroscopie, conflit, fémoro-acétabulaire, traitement, Arthroscopy, impingement, femoroacetabular, treatment

## Abstract

Le conflit fémoro-acétabulaire (CFA) est une des causes de douleur de hanche chez les sujets jeunes et sportifs. Son diagnostic est basé sur des arguments cliniques et radiologiques. La prise en charge chirurgicale peut être conventionnelle ou arthroscopique. L'arthroscopie s'avère être une technique fiable dans les CFA, efficace sur la douleur, permettant d'améliorer la fonction de ces patients sportifs afin de reprendre leurs activités précocement. Nous rapportons l'expérience de notre service dans la prise en charge arthroscopique des CFA à travers deux observations. Le score de Harris modifié était nettement amélioré au dernier recul (24 et 18 mois).

## Abstract (en)

Femoroacetabular impingement (FAI) can causes hip pain in young subjects and in athletes. His diagnosis is based on clinical and radiological examinations. Surgical treatment is based on conventional surgery or on arthroscopy. Arthroscopy proves to be a reliable technique in the treatment of FAI. It is effective on the pain and improves performances of athletes in order to enable them to resume their activities within a very short space of time. We here report our experience within our Department in treating 2 patients with FAI with arthroscopy. Modified Harris score significantly improved at the last follow-up (24 and 18 months).

## Introduction

Le CFA est un dysfonctionnement dynamique du à des chocs répétitifs, micro traumatiques entre la paroi acétabulaire et le col fémoral à sa jonction à la tête. Il est favorisé par une tête fémorale non sphérique, des parois acétabulaires trop couvrantes ou des mouvements excessivement amples responsables d'un excès de contraintes articulaires localisées qu'on retrouve notamment dans les sports de combat de pieds [1]. Deux types de conflits sont à distinguer: effet tenaille et effet came. Le traitement chirurgical des CFA est connu depuis longtemps [2], mais leur traitement arthroscopique a été rapporté pour la première fois en 1998, consistant en une résection ostéophytique [3]. Lorsque le démembrement des CFA fut établi par Ganz et al [4] en 2000, des méthodes arthroscopiques ont été proposées. L'efficacité de l'arthroscopie a été démontrée dans la prise en charge du CFA. C'est un traitement innovant, moins invasif, moins hémorragique, avec une meilleure exposition que la voie classique, et autorisant un levé précoce. C'est ainsi que nous avons voulu présenter l'expérience de notre service à travers deux observations cliniques.

## Patient et observation

**Figure 1:**
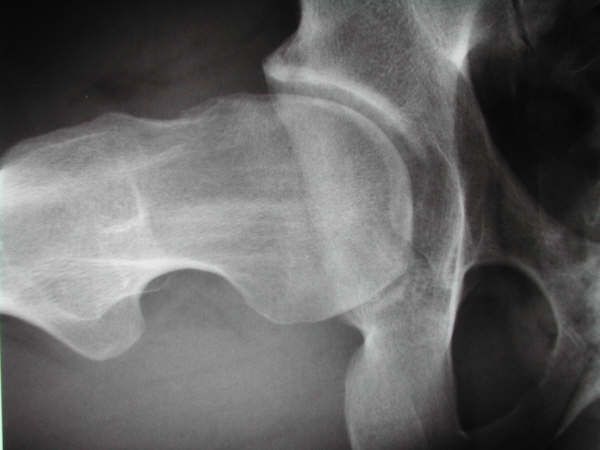
Radiographie de profil du col fémoral montrant une came fémorale chez le patient 1

**Figure 2:**
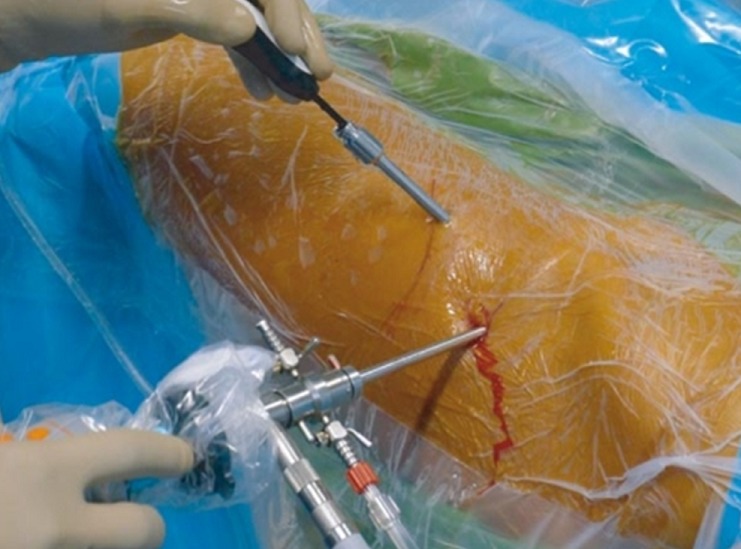
Voie d'abord arthroscopique de la hanche

**Figure 3:**
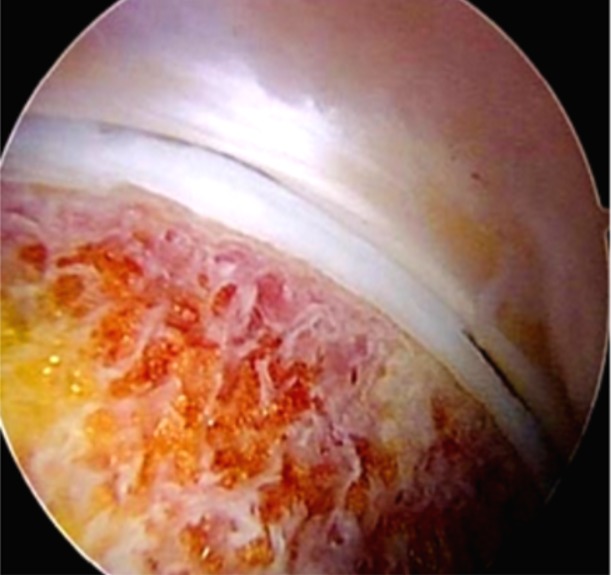
Aspect (arthroscopique) après résection de la came chez le patient 1

**Observation 1:** Il s'agissait d'un patient âgé de 27 ans, droitier de latéralité, pratiquant le Taekwondo depuis 10 ans, sans antécédents pathologiques notables, qui avait consulté pour une douleur de la hanche droite. La douleur était localisée au pli de l'aine, souvent ressentie dans la région fessière homolatérale, d'allure mécanique, d'abord insidieuse puis accompagnée de gêne fonctionnelle qui imposait la réduction de l'activité physique, associées à une sensation de craquement, évoluant depuis environ 10 mois. Cette douleur était reproduite lors d'une flexion et rotation interne de la hanche à l'examen. Ce dernier avait trouvé également une limitation de la rotation interne. Une radiographie du bassin de face en charge, permettait d'apprécié la couverture de la tête fémorale, l'interligne articulaire, l'antéversion et la profondeur du cotyle et de chercher un éventuel trouble de la version pelvienne. une incidence de profil du col fémoral (en incidence d'Arcelin), qui a objectivé une came fémorale (Figure 1) et sur laquelle nous avions mesuré l'angle alpha (permet d'évaluer l'importance de la came fémorale), correspondant à l'angle formé par la droite passant par l'axe du col fémoral et celle qui relie le centre de la tête fémorale au point où cette tête perd en avant sa sphéricité (alpha = 72°). L'arthro-scanner de la hanche avait confirmé la came fémorale, sans lésion du labrum. Ce bilan radiologique nous a permis de retenir le diagnostic de conflit par effet came dû à l'existence d'une lésion de type «Bump» sans atteinte du labrum. La prise en charge a été réalisée par arthroscopie. Après une anesthésie générale, le patient était installé en décubitus dorsal, sur une table orthopédique, avec une contre traction de la hanche sur un support périnéal large et mousse, stabilisant le bassin. Nous avions réalisé une première voie d'abord optique antérolatérale de la hanche, puis une seconde voie d'abord antérolatérale distale pour réaliser une capsulotomie antérieure. Une distraction de la hanche nous a permis de visualiser l'articulation et de faire un bilan intra-auriculaire. La came fémorale était bien visualisée . Une fémoroplastie a été réalisée chez ce patient. Elle a consisté en une correction de la came fémorale par résection de celle-ci (Figure 2) avec réalisation d'une échancrure du col fémoral (Figure 3). Le séjour était de 24 heures. Les suites opératoires étaient simples, avec douleurs modérées (EVA 4), levé le même jour. Le patient avait bénéficié d'une rééducation fonctionnelle. Le score modifié de Harris était nettement amélioré avec reprise d'activités au bout de trois semaines. Ce résultat très encourageant a été retrouvé au dernier recul de 24 mois.

**Figure 4:**
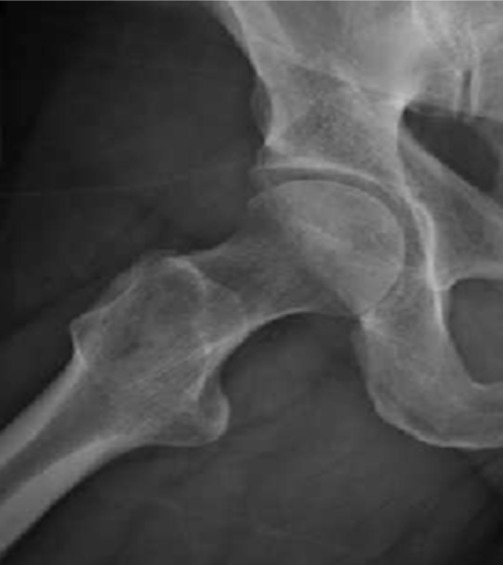
Aspect radiologique après résection de la came fémorale 3 semaines post opératoire chez le patient 1

**Observation 2:** Il s'agissait d'un patient âgé de 30 ans, footballeur, droitier de latéralité, sans antécédents pathologiques notables, qui avait consulté pour une douleur de la hanche droite. Il s'agissait d'une douleur au pli de l'aine, d'allure mécanique, évoluant depuis environ 8 mois avec réduction de l'activité physique. Une flexion de la hanche à l'examen clinique réveillait la douleur. Une radiographie de profil du col a objectivé une came fémorale et l'angle Alpha mesuré est de 68°. Arthro-scanne avait confirmé également la came fémorale, sans lésion du labrum. La prise en charge arthroscopique a été pratiquée selon les mêmes modalités décrites ci-dessus. La came fémorale était bien visualisée (**Figure 4**). Le patient avait séjourné 24h. Les suites opératoires étaient simples avec levé le même jour. Après quelques séances de rééducation fonctionnelle, il avait repris ces activités au bout de deux semaines. Là également le score modifié de Harris était nettement amélioré et ce résultat a persisté jusqu'au dernier recul de 18 mois.

## Discussion

Les orientations thérapeutiques sont encore récentes et non appuyées par des séries larges avec un grand recul. Elles doivent donc être interprétées avec prudence. L'objectif du traitement arthroscopique est double : l'amélioration des symptômes et la prévention de l'évolution arthrosique [4-6]. Chez les patients limités dans leurs activités professionnelles, la chirurgie doit être évoquée après 3 à 6 mois de traitement conservateur. Le traitement à ciel ouvert par luxation de la hanche trouve son intérêt dans l'exposition de la totalité de l'articulaire permettant une exploration. Ganz avait décrit en 2001 une technique sûre et efficace pour explorer toute la hanche par luxation sans risque de nécrose. Cette approche permettait le traitement de la totalité des lésions, à la jonction tête-col, du labrum, du cartilage qu'elles soient antérieures ou postérieures [7]. Lorsque le démembrement des CFA fut établi par Ganz et al [4] en début des années 2000 en distinguant les dysmorphies fémorales, acétabulaires et les formes combinées, des méthodes arthroscopiques ont été proposées. L'efficacité du traitement arthroscopique a été démontrée dans la prise en charge du CFA. Ce traitement arthroscopique permet entre des mains expérimentées la réalisation de tous les gestes thérapeutiques : ostéoplastie, débridement et réinsertion labrale. Les défenseurs de cette technique rapportent des résultats équivalents au traitement à ciel ouvert en l'absence d'arthrose [8, 9]. La principale complication redoutable de cette méthode bien que rare (2%) et transitoire, est selon certains auteurs la neurapraxie pupendale dont la survenue est liée au point d'appui périnéal [10]. Cette complication n'a pas été enregistrée chez nos patients du fait de l'utilisation d'un appui large et mousse, d'une durée de traction moindre.

## Conclusion

Les CFA sont une entité clinique d'identification récente. Leur traitement doit viser la correction des vices architecturaux là où ils siègent. Pour cela, le traitement arthroscopique constitue un consensus admis actuellement. Il s'agit d'une technique fiable, très innovante et prometteuse qui permet d'être efficace sur la douleur et d'améliorer la fonction de ces jeunes patients sportifs.

## Conflits d’intérêts

Tous les auteurs ne déclarent aucun conflit d'intérêts.
